# Supplementary material for: A city of cities: Measuring how 15-minutes urban accessibility shapes human mobility in Barcelona
Source: PLoS One. 2021 May 5;16(5):e0250080. doi: 10.1371/journal.pone.0250080 (PMC8099121; doi:10.1371/journal.pone.0250080)
Supplement: S1 Appendix — (PDF) [file pone.0250080.s001.pdf]

A city of cities: The influence of local amenities in shaping human mobility flows in Barcelona

Supporting Information

Eduardo Graells-Garrido[1,4,*], Feliu Serra-Burriel[1,2], Francisco Rowe[3], Fernando M. Cucchietti[1], Patricio Reyes[1]

1 Barcelona Supercomputing Center (BSC), Barcelona, Catalonia, Spain

2 Department of Statistics and Operations Research, Universitat Politècnica de Catalunya, Barcelona, Catalonia, Spain

3 Geographic Data Science Lab, Department of Geography and Planning, University of Liverpool, Liverpool, United Kingdom

4 Data Science Institute, Universidad del Desarrollo, Santiago, Chile

* eduardo.graells@bsc.es

A Visitor Influx in Barcelona

To explore how these visitors move in the city, we computed a daily influx score in the city for each type of visitor. The score provides the number of active events triggered by their mobile phones, and therefore it is a proxy for the number of people in the city at a given time (see Fig S1). The marked oscillations in this score correspond to the weekly cycles modulated by the presence of commuters and locals leaving Barcelona during weekends. Regarding special events and dates, we can identify the summer season and the influence of tourists, residents on summer holidays during August, Easter, and the Christmas break (usually between December 23 and January 6), as well as special events such as the Mobile World Congress in February 2018.

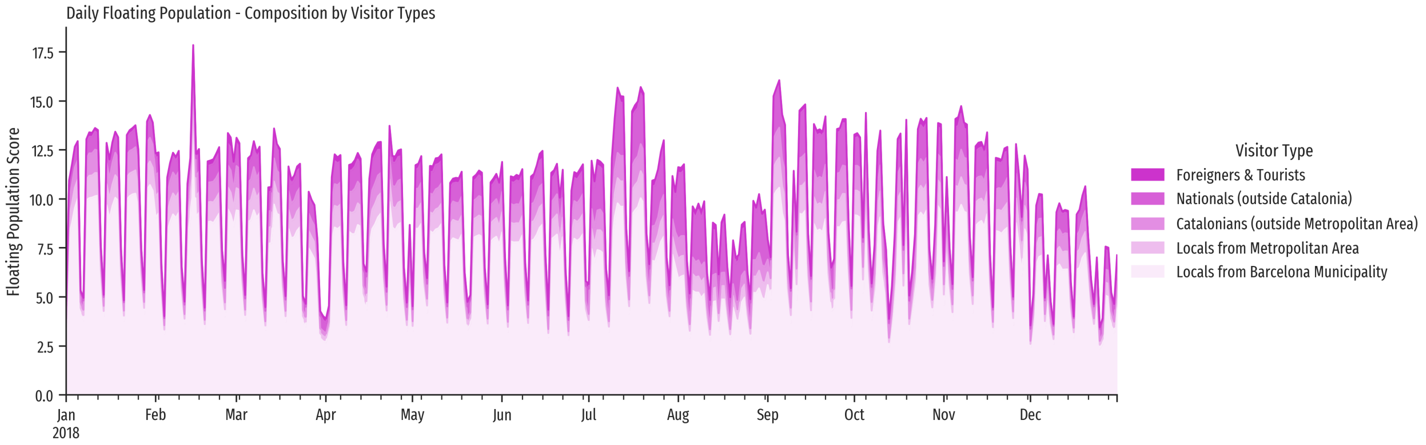

Fig S1. Daily population patterns in Barcelona. Stacked area time series of our daily floating population score in the municipality of Barcelona, categorized by origin type (i.e. users' usual address as inferred from their mobile phone). Note that the value being visualized is a floating population score, as the mobile phone data may contain differences in the several four-hour time frames of the day regarding active users. Here we focus on the analysis of mobility of city residents.

B Model Observations and Behavior

We analyze June as the most neutral month with respect to mobility within the city. Fig S2 shows the distribution of the mean visitor influx through the city, which was fitted using Negative Binomial (NB) regression in the global model, and NB Geographically Weighted Regression in the local model.

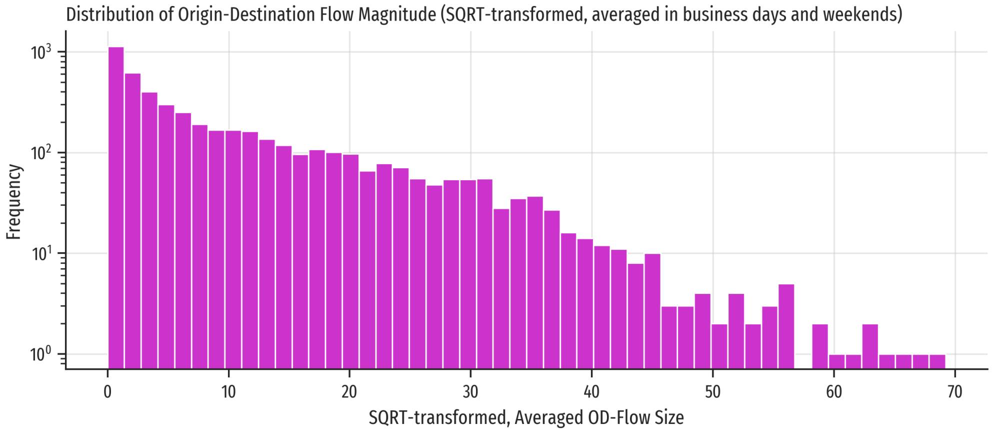

Fig S2. Origin-destination flow magnitude histogram (June 2018). Distribution of the transformed OD flow magnitude (square root of number of locals that visit a neighbourhood d that live in a neighbourhood o).

Fig S3 shows the Akaike Information Criterion corrected (AICc) resulting from applying the NB regression to each month. The only months with a better fit than June

are August and December, which have their own special characteristics that do not generalize to the rest of the year.

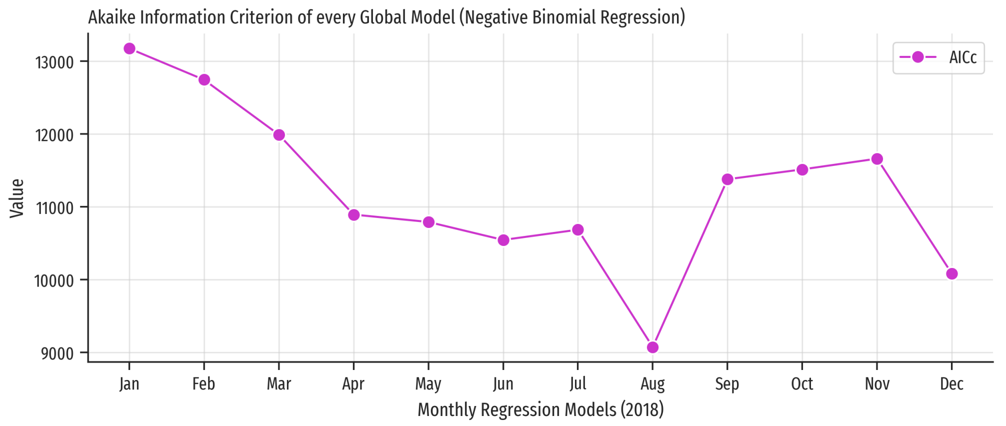

Fig S3. Akaike Information Criterion corrected (AICc) as model selection metric. We adjusted a global gravity model with Negative Binomial regression on the average flows of every month. A lower AICc value indicates less information loss in the model adjustment.

We also explored the residual distribution of both models in Fig S4. Note that the distributions are symmetric and centered around zero, signalling a well-behaved model.

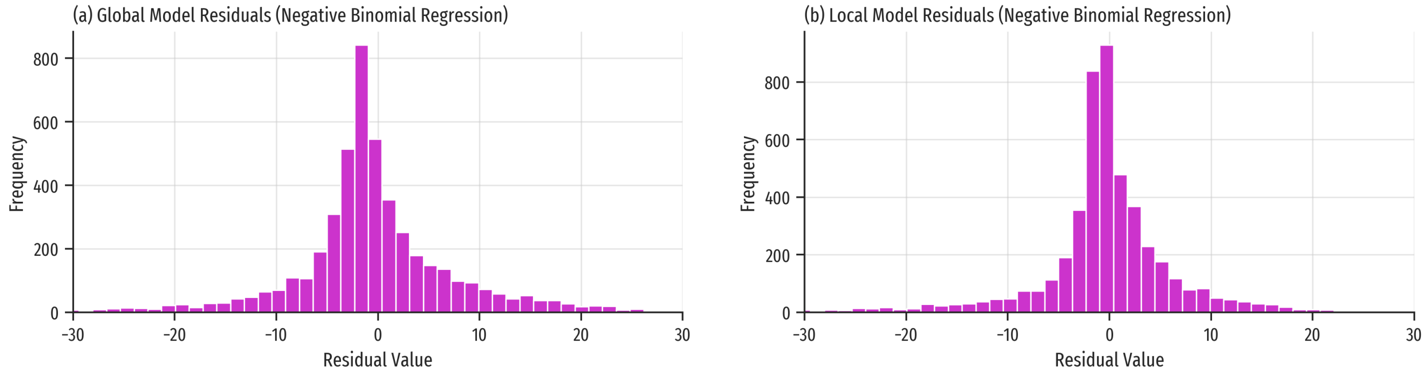

Fig S4. Distribution of model residuals, both global and local. a) Global model residuals. b) Local model (GWR) residuals.

C Geographical Variation of Local Factors

To explore the geographical variation of the local factors, we first estimated which local factors were significant using covariate-specific critical tests [1]. Few factors are significant in all neighborhoods (see Fig S5), and most are significant in less than half of neighborhoods. There are four factors that were completely non-significant:

Δ *government*, Δ HDI, Δ *age*, and Δ *immigrants*. The first may be explained due to government amenities being mainly available at the center of the city (see Fig **??**), whereas the other variables are of socio-demographic nature, hinting that people tend to visit neighborhoods with similar traits in that aspect. The variables that are significant in most neighborhoods either have all positive or negative factors for every neighborhood. These are log(*distance*), with all neighborhoods having significant factors, implying that distance is a relevant and negative factor for all; *in weekend*, with all neighborhoods having significant factors as well, implying that all neighborhoods reduce their mobility on weekends (or that some of them go outside the area of study); and lastly, *to other neighborhood*, with more than 90% of significant values, showcasing that the tendency to go to other neighborhoods is global.

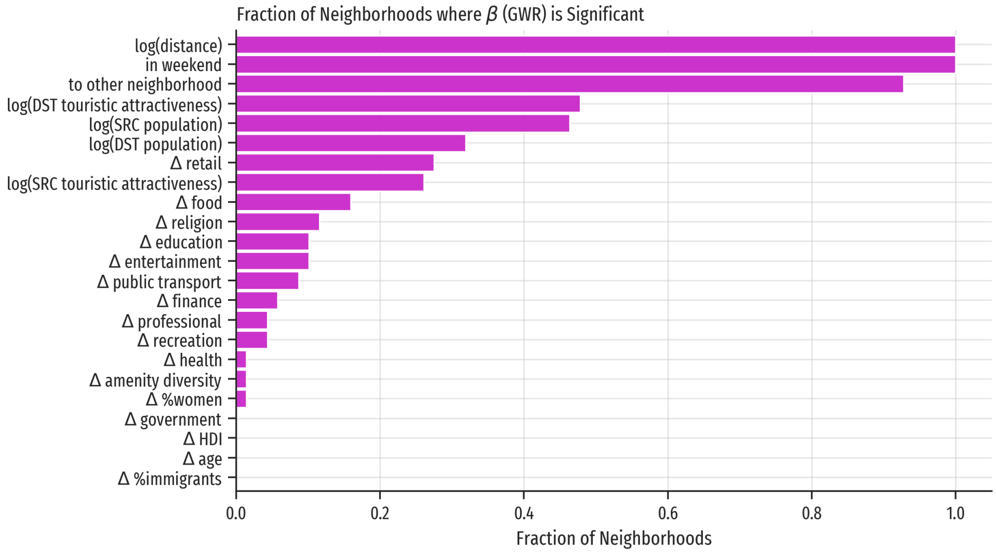

Fig S5. Local factors and the fraction of neighborhoods where these factors were significant. The significance of each local factor from the GWR model depends on the critical threshold determined by the distribution of all values and their covariation.

Next, we proceeded to compare how the local factors deviated from those in the global model. To do so, we compared the averages of each factor at each district of the city (the highest level in its administrative division), as well as every neighbourhood's own factor values to explore variability within districts. We found that even within districts there may be contrasting local factors from close neighbourhoods (see Fig S6), implying that district boundaries do not necessarily cluster accessibility and mobility

patterns. We also found that people living in central neighbourhoods of the city (such as the ones in the *Eixample* (IDs. 5, 6, 7, 8, 9, 10) and *Ciutat Vella* (IDs. 1, 2, 3, 4)) are the ones that have less tendency to move away from these districts, probably because they have access to all sorts of amenities and services, and thus, show smaller values in comparison to the rest of the city. Not only that, but also they probably have the best quality amenities, meaning that diversity and also the quality of these is important — this is a relevant aspect to include in future work. Furthermore, this suggests that there is a hierarchy within the different centers of the city, as the periphery small cities are more dependent on the mobility to other parts of the city to access the central amenities, which may be of better quality or have any other trait that makes them more attractive.

References

1. Oshan TM, Li Z, Kang W, Wolf LJ, Fotheringham AS. mgwr: A Python implementation of multiscale geographically weighted regression for investigating process spatial heterogeneity and scale. ISPRS International Journal of Geo-Information. 2019;8(6):269.

Comparison between β Coefficients in Local (GWR NB) and Global (NB) Models per District

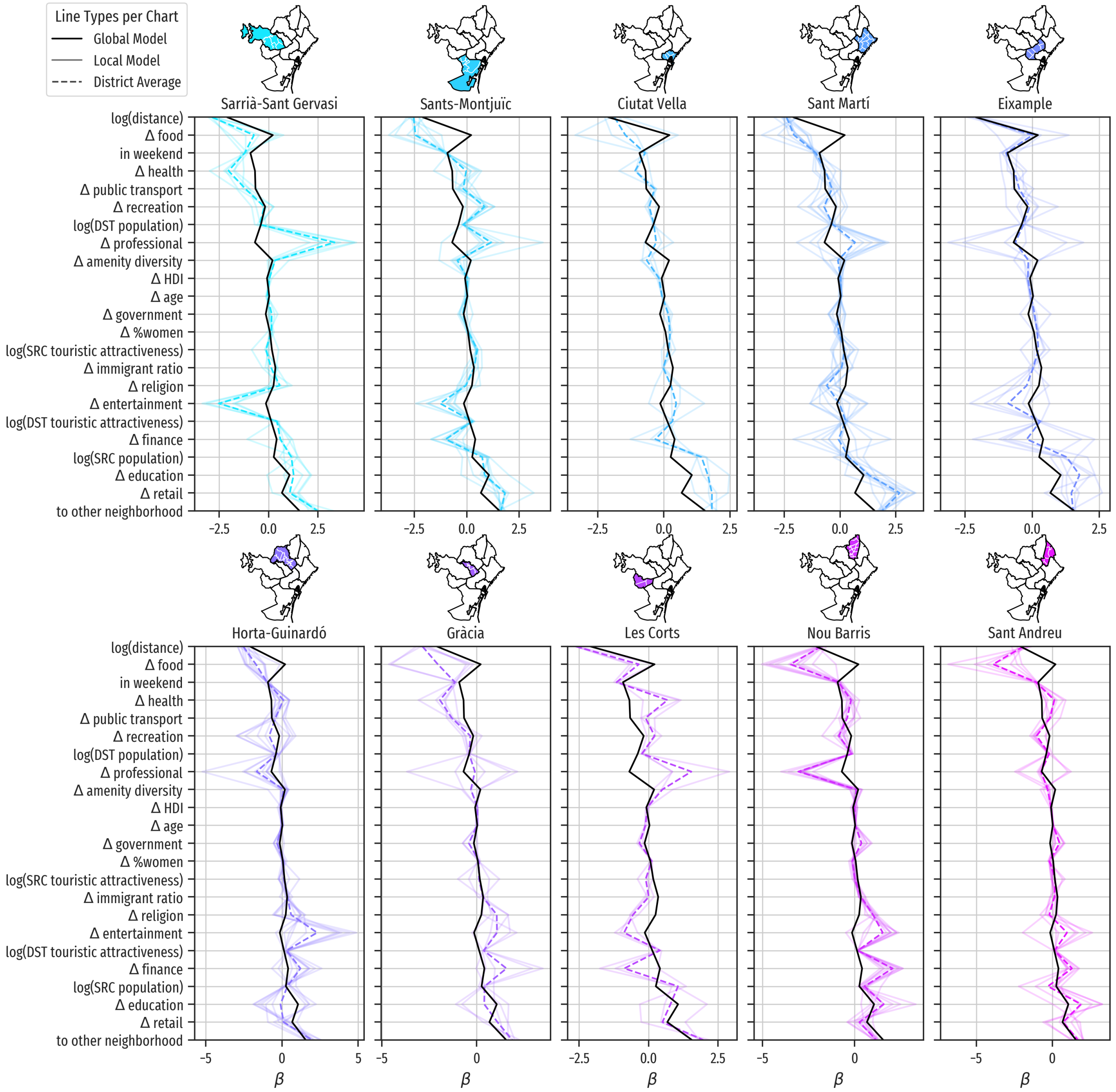

Fig S6. Parallel Coordinates plots of local model factors for each district. Every district is represented with a vertical line chart, where each colored line represents a neighbourhood within the corresponding district. Each line connects its corresponding local model factors. The global model beta values are plotted as black lines, and the mean values per district as dotted lines. Administrative boundaries are sourced from CartoBCN under a CC BY 4.0 license, with permission from Ajuntament de Barcelona, original copyright 2020.
